# Bispecific Antibodies Progression in Malignant Melanoma

**DOI:** 10.3389/fphar.2022.837889

**Published:** 2022-03-23

**Authors:** Juan Tang, Youling Gong, Xuelei Ma

**Affiliations:** Department of Oncology, West China Hospital of Sichuan University, Chengdu, China

**Keywords:** bispecific antibody, melanoma, intracellular target, immunotherapy, cytotoxicity

## Abstract

The discovery of oncogenes and immune checkpoints has revolutionized the treatment of melanoma in the past 10 years. However, the current PD-L1 checkpoints lack specificity for tumors and target normal cells expressing PD-L1, thus reducing the efficacy on malignant melanoma and increasing the side effects. In addition, the treatment options for primary or secondary drug-resistant melanoma are limited. Bispecific antibodies bind tumor cells and immune cells by simultaneously targeting two antigens, enhancing the anti-tumor targeting effect and cytotoxicity and reducing drug-resistance in malignant melanoma, thus representing an emerging strategy to improve the clinical efficacy. This review focused on the treatment of malignant melanoma by bispecific antibodies and summarized the effective results of the experiments that have been conducted, also discussing the different aspects of these therapies. The role of the melanoma epitopes, immune cell activation, cell death and cytotoxicity induced by bispecific antibodies were evaluated in the clinical or preclinical stage, as these therapies appear to be the most suitable in the treatment of malignant melanoma.

## Background

Melanoma is considered a multifactorial disease due to trauma, sunlight, biology, immunity, and genetic susceptibility. It has a predominant cutaneous localization, as compared with other cancers. Thus, early detection is still a key factor in reducing mortality (Lai et al., 2018). Melanoma spreads easily and metastasizes rapidly, and patients with metastases are almost always incurable disease, resulting in the death of most of these patients (Xie et al., 2022). The high risk of metastasis is the cause of the complexity of melanoma treatment.

Generally, melanoma is treated using comprehensive treatments. When detected at an early stage, the tumor can be removed by local resection, extensive local resection, and lymphadenectomy (Saltman et al., 2010). However, the effect of the surgical treatment is very limited in patients with advanced stages and tumor metastases, and comprehensive treatments including radiotherapy and drug therapy, are indispensable (Cherobin et al., 2018). Current chemotherapeutic drugs are of limited effect for most patients with advanced melanoma (Kozovska et al., 2016). Until recently, dacarbazine has been considered as the standard chemotherapeutic drug for metastatic melanoma, although the best effect is a partial remission (Koller et al., 2016). Driver mutations of melanoma often occur in the regulation of proliferation, metabolism, apoptosis and cell cycle control (Scolyer et al., 2011). BRAF and MAPK mutation are considered early carcinogenic events in melanoma (Davis et al., 2019). Striking BRAF inhibitors, including vemurafenib and dabrafenib, are effective in metastatic or advanced BRAF-mutated melanoma patients (Chapman et al., 2011). Although BRFA inhibitors are quite effective in BRAF-mutated melanoma patients, most of the patients develop a secondary resistance after an effective treatment. Then, immunomodulators emerged to treat melanoma, including IFN and IL-2, providing a promising approach for further research. One of the immunomodifiers developed for metastatic melanoma is interleukin 2 (IL2), which promotes the expansion of melanoma-specific T cells (Byrne and Fisher, 2017). In addition, the immunosuppressive molecules CD200 and immune checkpoint proteins expressed on melanoma cells, such as CTLA-4 and PD-L1, have also been identified as immunotherapeutic candidates (Talebian et al., 2012). Currently, three immune checkpoint inhibitors are used to treat melanoma, including the anti-CTLA-4 antibody ipilimumab and the two anti-PD-1 antibodies nivolumab and pembrolizumab (Topalian et al., 2015). Iplimumab treatment results in a long-lasting survival of up to 10 years in 20% of advanced melanoma cases, representing a great improvement compared to the median survival of less than 1 year in patients without immunotherapy (Sharma and Allison, 2015; Topalian et al., 2015). Although checkpoint inhibitors are promising treatments, the activation of the immune system surely causes a variety of side effects (Davis et al., 2019). In addition, the problem of immunotherapy resistance still needs to be resolved. Therefore, the exploration of new bispecific antibodies should be immediate.

## Basic Characteristics of Bispecific Antibodies

Although monoclonal antibodies are clinically effective in treating solid tumors and hematological malignancies, bispecific antibodies can have altered forms and binding part of the molecule to enable the combination of various anti-tumor functions. These alterations include changes in size, binding valence and geometric shapes. Changes in the binding part, including linker length or domain composition, may affect the function of the bispecific antibodies. According to different functional requirements, bispecific antibodies are designed to have different advantages in anti-tumor therapy (Lim et al., 2021). According to the assembly of bispecific antibody building blocks, the format of the molecule is divided into no Fc and Fc. Bispecific antibody fragments without Fc can easily combine multiple antibody fragments (Goebeler et al., 2016). However, these formats do not have Fc regions, their plasma half-life is very short and lacks Fc-mediated functions, including immune-mediated cell death. In addition, the lack of the Fc segment in the composition of bispecific antibodies may lead to a decreased stability of the antibodies and promote their mutual aggregation, thus affecting their biological functions (Kontermann and Brinkmann, 2015; Ayyar et al., 2016). In addition to the shortcomings mentioned above, they are simple in structure, small in size, with a good tissue permeability, and easy to produce (Labrijn et al., 2013). Bispecific antibodies with Fc structure have a long half-life in plasma and Fc-mediated cytotoxicity. The functions include complement-dependent cytotoxicity, Ab-dependent phagocytosis, and the induction of antibody-dependent cellular cytotoxicity. Another advantage is that its purification process is easy, with high stability and good solubility (Li et al., 2020). In conclusion, bispecific antibodies modified by different methods can achieve specific useful functions, including the regulation of immunogenicity, effector function and antibody half-life. Bispecific antibodies also kill tumor cells expressing specific targets, including internal tumor antigens and increase the number of effector T cells in tumor lesions (Weisbart et al., 2012; Ma et al., 2019; de Jong et al., 2021).

Thus, bispecific antibodies have a great potential in the treatment of melanoma, and large clinical trials with enough data are needed to clarify the best position of these drugs in the treatment paradigm and their effects on patients at an advanced stage of melanoma. The checkpoint inhibitors of melanoma, T cell co-stimulation, the target of multiple melanoma epitopes and melanoma intracellular targets are interesting areas that require further research in bispecific antibodies. Thus, this review explores the current status and future treatment prospects of bispecific antibodies in melanoma. Bispecific antibodies are divided into four groups based on the mechanism of action: 1) Tumor-targeted immunomodulators, 2) tumor-targeted death, 3) engaging immune cells and 4) regulation of intracellular targets.

**FIGURE 1:**
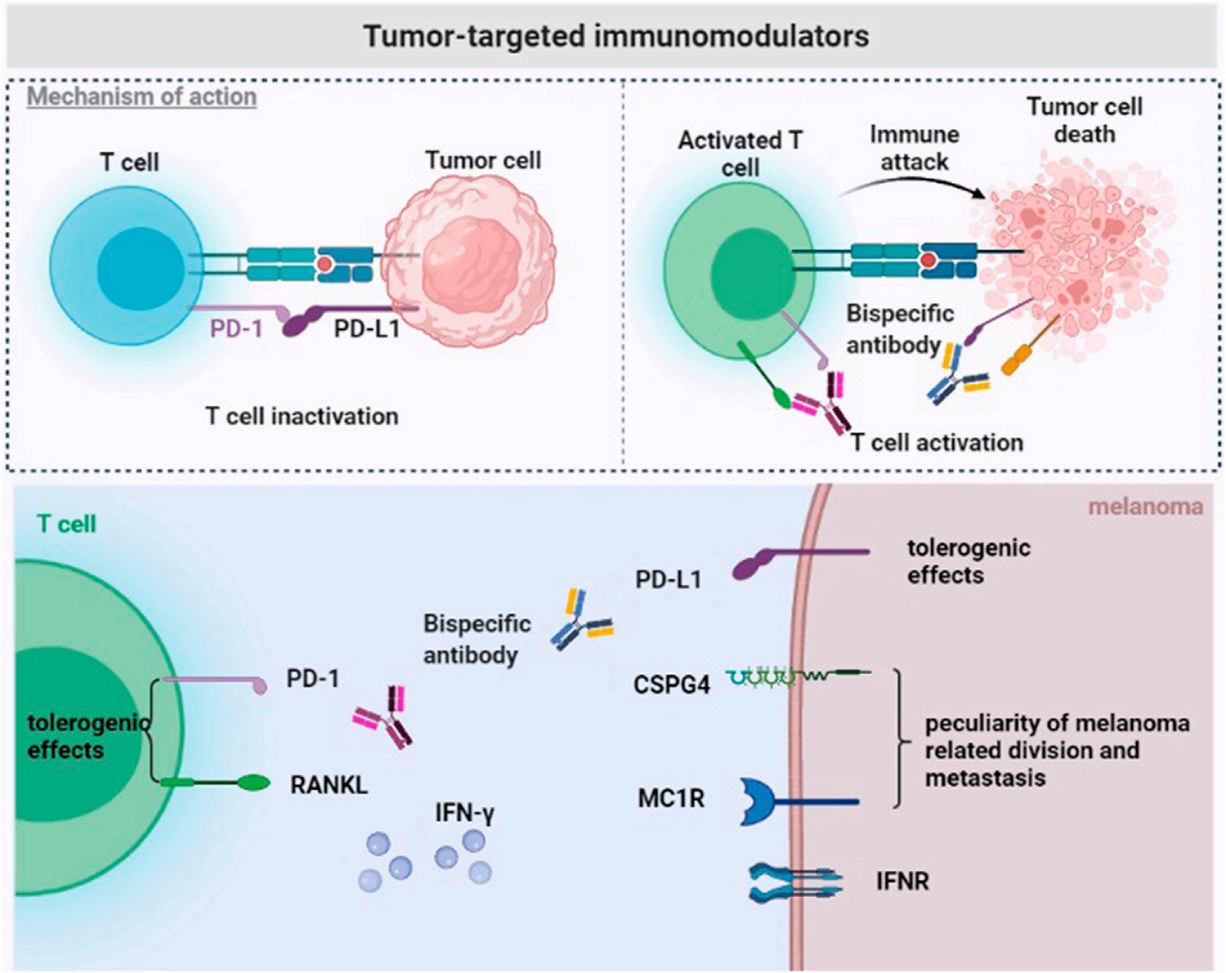
Tumor-targeted immunomodulators. The immunosuppressive tumor microenvironment of melanoma. Bispecific antibodies exert anti-tumor effects by binding to tumor-specific antigens (CSPG4/MC1R) and PD-L1, or to double immune checkpoints (RANKL and PD-1) on the surface of T cells.

**FIGURE 2:**
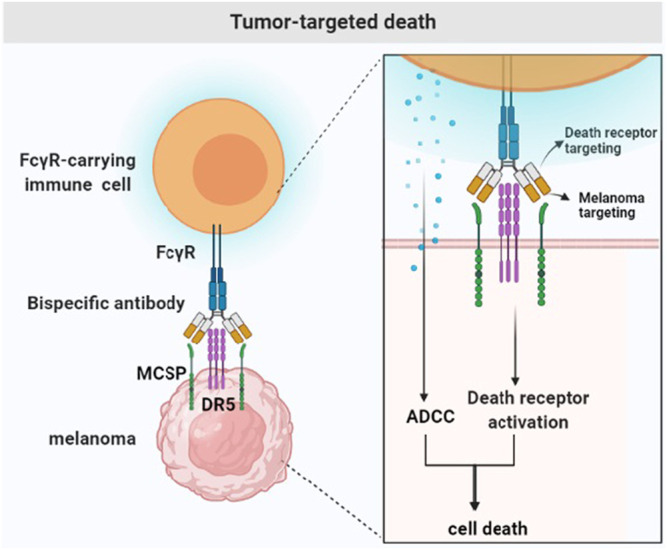
Tumor-targeted death. Tumor drug resistance is due to the prevention of the initiation or execution of the cell death signal. Bispecific antibodies bind to the tumor-specific antigen (MCSP) to activate the death signaling pathway (DR5) and Fc*γ*R-carrying immune cells.

**FIGURE 3:**
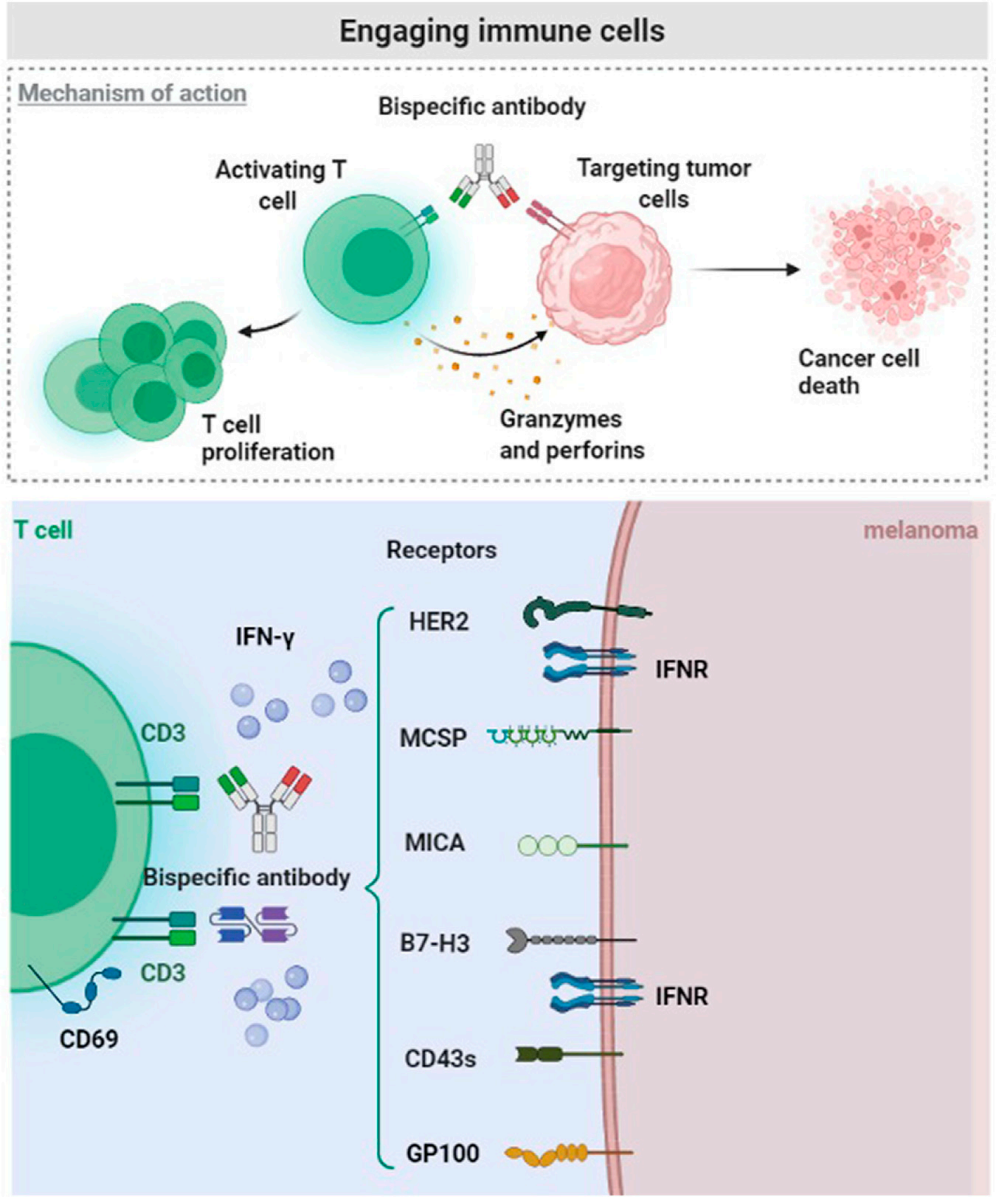
Engaging immune cells. The malignant melanoma microenvironment hinders the transport, infiltration and activation of T cells. CD3 related Bispecific antibodies bind tumor-specific antigen (HER2, MCSP, MICA, B7-H3, CD43s, and Glycoprotein 100), potentially promoting the intratumoral infiltration and response of T cells.

**FIGURE 4:**
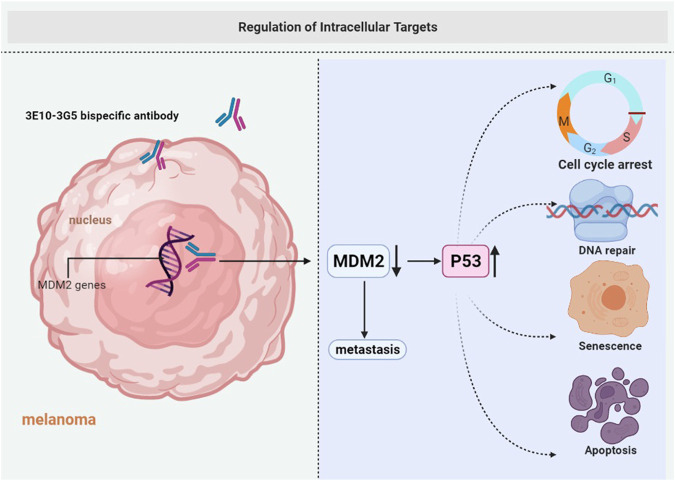
Regulation of intracellular targets. Cell membranes are impermeable to most macromolecules. 3E10-3G5 bispecific antibody retains cell-penetrating and MDM2-binding activities, increases tumor p53 expression, and inhibits the growth of tumors.

Potential targets and current antibodies under development are summarized in Figures 1–4 and Table 1.

**TABLE 1:** Melanoma cell related markers in bispecific antibody pre-clinical trials.

Antigen	Structure	Expression	Role	Construct	Mechanism of action
CSPG4	Consist of 280 kDa glycoprotein and 450 kDa chondroitin sulfate proteoglycan	Melanoma, mesothelioma, TNBC, Glioblastoma (Rivera et al., 2012)	Improve the selectivity, effectiveness and safety of checkpoint blockade	Anti-CSPG4 x anti-PD-L1 (Koopmans et al., 2019)	Tumor specific antigen
MC1R	Melanocortin receptor group	More than 80% of melanomas (Rosenkranz et al., 2013)	Improve the selectivity and increase cytotoxicity	Anti-MC1R x anti-PD-L1 (Zhang et al., 2019)	Tumor specific antigen
RANKL	The closest homology to CD40 and CD40L	CD8^+^, CD4^+^ T cells, tumor cells (Ahern et al., 2018a)	Enhance the efficacy of ICI	Anti-RANKL x anti-PD-1 (Liede et al., 2018)	Remove tolerogenic effects
DR5	Tumor necrosis factor related apoptosis-inducing ligand receptor	Highly expressed in melanoma cells (Leverkus et al., 2000)	Effectively and selectively kill melanoma cells	Anti-DR5 x anti- MSCP (Szegezdi and Leverkus, 2016)	Effective activation of DR5-mediated cell death signals
MCSP	Same as CSPG4	94% of MCSP expression is positive overall	Improve the selectivity, effectiveness and safety of BTiE	Anti-CD3 x anti- MSCP(Torisu-Itakura et al., 2011)	Specific activation of T cells and lysis of MCSP positive melanoma cells
B7-H3	Belongs to the B7/CD28 immunoglobulin superfamily (Steinberger et al., 2004)	Higher in a variety of human malignancies, including melanoma (Picarda et al., 2016)	Related proliferation, apoptosis and metastasis, tumor associated antigen	Anti-CD3 x anti-B7-H3 (Ma et al., 2019)	Transform immune evasion and generated higher levels of IFN-*γ*
CD43s	A sialylated variant of CD43, rich in serine and threonine residues (Tuccillo et al., 2014)	Present on lung, breast, cervical, colon, bladder, pancreas carcinoma and melanoma (Batdorf et al., 2017)	Related proliferation and extravasation tumor associated antigen	Anti-CD3xanti-CD43s (de Jong et al., 2021)	Induce T cells to kill melanoma cells
HER2	The epidermal growth factor receptor family	Various tumors (Bodey et al., 1997)	Related progression and metastasis, tumor associated antigen	Anti-CD3 x anti-HER2 (Ma et al., 2013)	Trigger T cell activation and cytokine secretion
MICA	One of the ligands of NKG2D	Tumors of the genitourinary system, gastrointestinal tract, and respiratory system, and melanoma (Shi et al., 2014)	Recognize the oncogenic transformation of cells	Anti- CD3 x anti-MICA (Godbersen et al., 2017)	Trigger activate T cells to kill tumor cell lines
Glycoprotein 100	100 kDa glycosylated transmembrane protein for melanocytes	Normal melanocytes and melanoma cells (Martinez-Perez et al., 2021)	Melanoma associated antigen	Anti-CD3 x anti-Gp-100 (Middleton et al., 2020)	Creating an immune synapse that kills targeted tumor cells
MDM2	An E3 ubiquitin ligase	Numerous malignancies, including melanoma (Weisbart et al., 2012)	Inhibition of MDM2 increases active p53 levels and induction of senescence in melanoma	3E10-3G5 bispecific antibody	Cell-penetrating and up-regulate the function of p53 (Verhaegen et al., 2012)

CSPG4, Chondroitin sulfate proteoglycan 4, also known as MCSP; MC1R, melanocortin 1 receptor; RANKL (TNFSF11), tumor necrosis factor receptor and ligand; DR5, death receptor 5; MCSP, melanoma-associated chondroitin sulfate proteoglycan; B7-H3 (CD276), the B7/CD28 immunoglobulin superfamily; CD43s, a sialylated variant of CD43; HER2, Human epidermal growth factor receptor-2; MICA, major histocompatibility complex class I-related chain A.

### Tumor-Targeted Immunomodulators

Although the current PD1/PD-L1 blocking antibody approved by the FDA shows significant therapeutic effects, especially in patients with melanoma and non-small cell lung cancer, these antibodies lack tumor-specific selectivity. Therefore, they arbitrarily activate T cells, including autoreactive T cells that are harmful to the body.

Chondroitin sulfate proteoglycan 4 (CSPG4), a type 1 transmembrane protein, is composed of a 280 kDa glycoprotein and a 450 kDa chondroitin sulfate proteoglycan and is also called MCSP. CSPG4 is highly expressed in various refractory malignant tumors, such as triple-negative breast cancer, glioblastoma, mesothelioma, and melanoma (Wang et al., 2010; Svendsen et al., 2011; Rivera et al., 2012). A study reported that CSPG4 is overexpressed in more than 90% of melanoma lesions and is not present in healthy adult tissues. Therefore, it represents a significant target antigen to combat malignant tumors. The bispecific antibody PD-L1xCSPG4 may improve the selectivity, effectiveness and safety of the therapies against malignant tumors overexpressing CSPG4, including melanoma. Importantly, some results indicate that the bispecific antibody PD-L1xCSPG4 induces T cell activation by targeting the CSPG4 antigen. In this case, PDL1xCSPG4 enhances the ability of T cells to secrete IFN–*γ*, thus exerting an anti-tumor effect (Koopmans et al., 2019) (Figure 1).

A report shows that the bispecific antibody avelumab composed of a melanocyte stimulating hormone (*α*-MSH) analog and PD-L1 antibody inhibits checkpoints on the tumor microenvironment and prevents systemic immune activation. Melanocortin 1 receptor (MC1R) is a marker of the risk of melanoma overexpressed in more than 80% of melanomas (Rosenkranz et al., 2013). It belongs to the melanocortin receptor group and binds to *α*-MSH to cause a biological response. The high MC1R expression not only represents a molecular characteristic of melanoma, but also has important implications for tumor cell division and metastasis. The bispecific antibody avelumab composed of *α*-MSH analog and PD-L1 antibody may exert a cytotoxic effect, reverse the immune suppression in the tumor environment, and reduce side effects (Zhang et al., 2019) (Figure 1).

Bintrafusp alfa works by targeting PD-L1 through IgG, and the peptide chain ends are fused with the extracellular domain of two TGF-*β* receptor II molecules, designed to “capture TGF-*β*” in the tumor microenvironment. According to the results of phase I clinical trials, bispecific antibodies are similar to other checkpoint inhibitors in safety but they have a better objective and effective clinical remission rate (Lind et al., 2020).

RANK (TNFRSF11a) and RANKL (TNFSF11) belong to the tumor necrosis factor superfamily (Anderson et al., 1997). Recently, the RANK signal in bone marrow cells is considered as tolerogenic under various conditions (Williamson et al., 2002; Demoulin et al., 2015; Fujimura et al., 2015). This suggests that RANKL/RANK antagonists can be potentially used as an anti-tumor therapy through immune activation. In addition, RANK and RANKL are overexpressed in tumor cells and different immune cell types in the tumor microenvironment. RANK is overexpressed in tumor infiltrating bone marrow cells, and RANKL is overexpressed in effector T cells and tumor cells (Lacey et al., 2012; Ahern et al., 2018a). RANKL/RANK blockade induces tumor- associated myeloid cell suppression and increases the number of tumor infiltrating lymphocytes to reverse T cell dysfunction in the tumor microenvironment. According to other reports, the simultaneous effect of anti-RANKL and immune checkpoint blockers enhances the anti-tumor effect in various mouse tumor types (such as prostate, fibrosarcoma and melanoma). This treatment method controls subcutaneous tumor growth or metastasis and, more importantly, in an anti-PD-1 resistant environment (Ahern et al., 2017; Bakhru et al., 2017; Ahern et al., 2018b). The superior therapeutic effect of the bispecific antibody anti-RANKL/PD-1 compared with the simultaneous action of two antibodies anti-RANKL and anti-PD-1 may be due to better biodistribution of anti-RANKL/PD-1, which the bispecific antibody to elevated levels of RANKL and PD-1 expressed in the TME, including, but not limited to, co-expression of both target antigens on CD8+TILs. The increased affinity obtained by the simultaneous co-target of two antigens may lead to a more precise targeting of tumors and a greater degree of immune checkpoint blockade. In addition, “real world” clinical evidence derived from case reports and retrospective clinical analysis of patients with advanced melanoma shows that the simultaneous inhibition of RANKL and PD-L1 promotes the efficacy of immune checkpoint inhibitors (Liede et al., 2018). Anti-RANKL/PD-1 especially shows an effective inhibition of tumor growth in an immune checkpoint inhibitor resistant environment, providing a new treatment strategy against tumors with advanced drug-resistance (Dougall et al., 2019) (Figure 1).

### Tumor-Targeted Death

An important factor leading to tumor resistance is the inhibition of the activation or conduction of cell death signals. Therefore, drugs need to induce cell death to destroy drug resistance, especially in cohorts of patients with poor prognosis (Hanahan and Weinberg, 2011). Death receptor-5 (DR5) is overexpressed in melanoma cells and activates cell death signals by binding to death ligand cytokines. Tumor necrosis factor (TNF), tumor necrosis factor-related apoptosis-inducing ligand (TRAIL) and CD95 ligand provides a direct induction of cell death. Among these death ligand cytokines, TRAIL has attracted considerable interest because of its specificity for transformed cells (Leverkus et al., 2000). TRAIL represents an attractive treatment for receptor-mediated apoptosis. It has attracted considerable attention due to its selective killing of tumor cells (Pan et al., 1997) and induction of apoptosis when binding to its own receptor, because TRAIL provides a strategy to directly trigger cell death. According to reports, the bispecific, tetravalent antibody MCSPxDR5 targets MCSP and simultaneously activates death receptor 5 (DR5, TRAIL-R2). The bispecific antibody is specific to MCSP-positive melanoma cells and activates the DR5-dependent death signaling pathway and exerts a cytotoxic activity. The cross-linking of antibodies to Fc*γ* receptors further increases the cytotoxic potential without affecting the selectivity. This method uses three coordinated processes: The binding of death receptor agonists in malignant tumor cells, the effective activation of cell death signals, and the recruitment of immune cells. Therefore, MCSPxDR5 can guide the anti-DR5 apoptotic function on melanoma cells, effectively and selectively killing melanoma cells expressing MCSP and DR5 (Szegezdi and Leverkus, 2016) (Figure 2).

Most of the therapeutic antibodies targeting ovarian cancer are largely disappointing due to the limited cytotoxicity of tumor-specific antibodies. Bispecific targeted cytotoxicity activator specifically binds to folate receptor *α*-1 (FOLR1) and DR5 to achieve “cis” and “trans” cytotoxicity. The DR5 signaling pathway is activated *in vivo* by interacting with Fc*γ*RIIB, and FOLR1 anchoring represents the basis in the maintenance of high levels of tumor-specific cell apoptosis (Shivange et al., 2018).

### Engaging Immune Cells

The tumor microenvironment in solid tumors hinders the transport and infiltration of T cells. CD3 related bispecific antibodies may promote T cell infiltration and HLA-independent T cell activation in tumors. All T cells express the CD3 antigen on the surface.

Many experiments proved that T cell-based therapies produce partial or complete responses to advanced melanoma, including T-cell gene therapy and adoptive T-cell therapy (Berry et al., 2009). MCSP expression varies in different melanoma cell lines, but 94% of MCSP expression is positive overall. The expression of MCSP antigen in melanoma cells is a promising target for immunotherapy, according to the characteristics of bispecific T cell junction (BiTE) (Torisu-Itakura et al., 2011). The tumor-specific antigen binds to one arm, and the CD3 subunit of T cell receptor binds to the other arm of the bispecific antibody, effectively stimulating T cell activation. Therefore, BiTE antibody directly activates T cells and does not require dendritic cells to produce specific T cell clones or antigen presentation (Baeuerle et al., 2009; Schmidt et al., 2011). MCSP-BiTE antibody co-cultured with peripheral mononuclear blood cells or CD8^+^ T cells mediates the lysis of MCSP positive melanoma cells (Torisu-Itakura et al., 2011) (Figure 3).

Immune checkpoint blockade achieved compelling results in malignant tumors and shows broad prospects (Larkin et al., 2015). B7-H3 is another immune checkpoint, and it is a member of the B7/CD28 immunoglobulin superfamily, also known as CD276. It is a type I transmembrane glycoprotein and a promising immunosuppressant (Steinberger et al., 2004). Importantly, B7-H3 is an inhibitory receptor for T cell activation and it is less expressed in healthy tissues, but overexpressed in various human malignancies (Wang et al., 2014; Picarda et al., 2016). In addition to the promotion of immune evasion by the tumor, B7-H3 is also related to tumor proliferation, apoptosis, adhesion, angiogenesis, invasion and metastasis (Castellanos et al., 2017). The overexpression of B7-H3 in melanoma is negatively related to the survival rate of patients, it participates in many metastatic-related pathways, and promotes tumor progression (Tekle et al., 2012; Wang et al., 2013). Studies confirmed that B7-H3 is a promising target for the activation of T cell immunotherapy. One arm of B7-H3 bispecific antibody recognizes tumor-specific antigens and the other arm binds immune cells. B7-H3 bispecific antibody activated T cells and produce higher levels of IFN-*γ* when interacting with melanoma cells, consequently exerting a tumor-killing effect (Ma et al., 2019) (Figure 3).

CD43 is a mucin-like transmembrane protein composed of serine and threonine residues (Tuccillo et al., 2014). It is considered as a marker of hematopoietic cells and is highly expressed in acute myeloid leukemia and myelodysplastic syndromes (Rosenstein et al., 1999). It is also highly expressed in various solid malignant tumors, and it is related to tumor promotion (Balikova et al., 2012; Batdorf et al., 2017). More importantly, CD43s is overexpressed in melanoma cell lines and patients’ melanoma samples. The synthesis of the bispecific T-cell activator based on AT1413 and CD3 where AT1413 is a ligand for CD43s is now possible. Two functional T-cell engager forms of AT1413 were synthesized, and the dual bivalent b T-cell engager format is approximately 50 times more effective than the unit price KiH format. These T-cell engagers effectively activate T cells to kill melanoma cells (de Jong et al., 2021) (Figure 3).

The epidermal growth factor receptor 2 (HER2), also known as erbB2, belongs to the epidermal growth factor receptor family. HER2 is highly expressed in various malignant tumors, such as female reproductive system malignancies and digestive tract malignancies. Many studies demonstrated that HER2 is expressed during the progression and metastasis of melanoma while it is not expressed in normal melanocytes. According to reports, HER2 was detected in 10 metastatic melanoma patients, and 8 of them showed a high expression (Bodey et al., 1997). A preclinical study evaluates the specific cytotoxic effect of Malme-3M-luc cells by anti-CD3 x anti-HER2 bispecific antibody (HER2Bi-Ab). High levels of the activation marker CD69 and secretion of considerable higher levels of IFN-*γ* are produced by HER2Bi-armed ATC. Therefore, HER2Bi-Ab not only binds to tumor-specific antigens, but also activates T cells and promotes cytokine secretion (Ma et al., 2013) (Figure 3).

Claireet al. developed and characterized a novel BiTE molecule specific for the NKG2D ligand. NKG2D plays a role in recognizing the oncogenic transformation of cells (Lanier, 2015). The expression of NKG2D ligand is controlled at the transcription, translation and post-translational levels. The expression of NKG2D ligand on the cell surface increases when the cell is under infection, transformation or other stress state (Del Toro-Arreola et al., 2011). Major histocompatibility complex class I-related chain A (MICA) is one of the ligands of NKG2D and is widely expressed on many malignant tumors, including those of the genitourinary system, gastrointestinal tract, and respiratory system. MICA is overexpressed in 75% of skin melanoma cases and 50% of metastatic melanoma cases (Shi et al., 2014). It becomes promising targets for immunotherapy because their expression in normal tissues is not abundant. T cells anchor tumors with high expression of MICA. Therefore, B2-OKT3 BiTE can target MICA on tumor cells and CD3ε on T cells through the tandem scFv BiTE format, which specifically induces T cells to kill tumor cells (Godbersen et al., 2017) (Figure 3).

Uveal melanoma is a disease different from skin melanoma. The 1-year overall survival rate for patients with metastatic uveal melanoma is approximately 50%. Glycoprotein 100 (Gp-100) is a 100 kDa molecule specific for melanocytes of the skin, mucosa and retina. It is a glycosylated transmembrane protein involved in melanosome maturation, which are organelles that transport melanin and they are highly expressed in normal melanocytes and melanoma cells (Middleton et al., 2020; Martinez-Perez et al., 2021). Different approaches for treating melanoma with Gp-100 as an antigen have been developed, including Gp-100-based vaccines, mRNA electroporation of dendritic cells, and fusion proteins named IMCGp-100 or tebentafusp. Tebentafusp is a first-in-class anti-Gp-100 immune mobilizing antibody. This bispecific fusion protein composed of a soluble affinity-enhanced TCR and an anti-CD3 scFv, targets tumor cells that express a peptide of Gp-100 presented by HLA*A0201, creating an immune synapse that kills targeted tumor cells (Middleton et al., 2020). Tebentafusp treatment used in a randomized phase 3 clinical trial was positively correlated with the overall survival of metastatic uveal melanoma (Nathan et al., 2021) (Figure 3).

Currently, CD3-related bispecific antibodies are already used in clinical or preclinical trials of other tumors. The treatment options for HER2-negative breast cancer are limited. Phase I studies showed that anti-CD3 x anti-HER2 bispecific antibodies (HER2 BATs) activate T cells, representing a promising approach in the treatment of HER2-positive patients. A phase II trial evaluated the efficacy and immune response of HER2 BAT as an immuno-consolidation therapy after chemotherapy in HER2-negative patients. The use of HER2 BAT for immune consolidation after chemotherapy in a number of treated HER2 patients increases the number of stable patients and the median overall survival, as well as the adaptive and innate anti-tumor response (Lum et al., 2021). B cell maturation antigen is an effective target for relapsed or refractory multiple myeloma. Teclistamab binds to B cell maturation antigen and CD3 to form a bispecific antibody that redirects T cells to multiple myeloma cells. At the recommended dose in the phase 2 clinical trial, Teclistamab was maintained above the target dose level to achieve a consistent T cell activation. It shows high safety, good tolerability and curative effect in patients with relapsed or refractory multiple myeloma (Usmani et al., 2021).

### Regulation of Intracellular Targets

Antibodies play an important role in tumor therapy, but their use in targeting intracellular tumor antigens is difficult. Cell membranes are impermeable to most macromolecules, thus, the modulation of intracellular target antigens is primarily based on the use of small molecules that can passively diffuse into cells. However, small molecule inhibitors are prone to off-target effects, leading to a significant toxicity (Zhang et al., 2009). Several different strategies have been explored to facilitate the passage of macromolecular proteins across cell membranes, including electroporation, microinjection, cell penetrating peptides, and liposomes (Singh et al., 2019).

In addition to the above-mentioned, a cell-penetrating monoclonal antibody 3E10 was developed as an intracellular delivery vehicle for the intracellular and intranuclear delivery of antibodies to overcome the limitation of intracellular targets. It is a rare monoclonal anti-DNA antibody that penetrates living cells and localizes in the nucleus without causing any appreciable damage to the cells (Hansen et al., 2007a). The monoclonal antibody 3E10 and its single-chain variable fragment (3E10 scFv) have been developed as intracellular delivery systems for macromolecules (Weisbart et al., 2004; Hansen et al., 2007b). The inhibition of MDM2 in melanoma immunotherapy increases active p53 expression and is associated with the death of human melanoma cell lines (Verhaegen et al., 2012). Since MDM2 is an important target in melanoma, the monoclonal antibody 3G5 is chosen for intracellular transport. The mAb 3G5 binds MDM2 and blocks MDM2 binding to p53. The 3E10-3G5 bispecific antibody retains cell-penetrating and MDM2-binding activities, increases tumor p53 expression, and inhibits the growth of MDM2-associated tumors (Weisbart et al., 2012) (Figure 4).

Immunotherapy is a promising chemotherapy against resistant leukemia. However, it is limited by the ability of targeting lineage-specific cell surface antigens. A large number of leukemia-related targets become accessible by targeting intracellular antigens. A novel T-cell bispecific (TCB) antibody was generated using CrossMab and Knob-in-pore technology, containing a bivalent T-cell receptor-like binding domain that recognizes RMFPNAPYL derived from intracellular tumor antigens peptide in the context of human leukocyte antigen (HLA) A*02, Wilms’ tumor 1 (WT1). The binding to CD3ε leads to the recruitment of T cells regardless of T cell receptor specificity. WT1-TCB promotes an efficient *in vitro*, *ex vivo* and *in vivo* killing of acute myelocytic leukemia cell lines and primary acute myelocytic leukemia. These results lead to the initiation of a phase I trial in patients with r/r AML (Augsberger et al., 2021).

## Conclusion

Clinicians still face great challenges when faced with malignant melanoma, especially in patients with advanced cancer. Immunotherapy has improved the prognosis of melanoma over the past decade. Monoclonal antibodies exert their effects through various mechanisms of action (Redman et al., 2015). For example, antibodies directly block the binding of angiogenic factors to their receptors, or block cell mitosis through membrane tyrosine kinases. In addition, antibodies can stimulate the immune system of the patient and exert cytotoxic effects on the tumor, including antibody-dependent cellular phagocytosis, antibody-dependent cytotoxicity, and complement-mediated cytotoxicity (Agus et al., 2002; Strome et al., 2007; Sullivan and Brekken, 2010; Azoury et al., 2015). However, monoclonal antibodies used in some patients have been found as ineffective or inducing the progression of the disease. Thus, more effective treatment methods should be explored. The concept of bispecific antibodies first appeared in the 1980s (Raso and Griffin, 1981), and they can realize multiple antitumor functions among the above-mentioned regarding monoclonal antibodies. Bispecific antibodies have the specificity of two antibodies; thus, they can bind tightly to two different target molecules or cell types (Szegezdi and Leverkus, 2016). Adaptive immunity needs to initiate an antigen-specific response. The key step in this process is to activate T cells, which recognize and kill antigen-specific cells. Antigen presenting cells capture and process antigens, then enter the lymph nodes and present the processed antigens to major histocompatibility complex I/II antigen-specific T cell molecules. The complex T cell activation process leads to immune inactivation, resulting in the immunosuppression of the tumor microenvironment. The CD3 subunit is a protein for T cell activation, and it does not use cell antigen presentation of antigen presenting cells to simplify T cell activation. The bispecific antibody activates T cells while producing cytotoxicity through this mechanism. Blinatumomab (CD3 ×B lymphocyte antigen CD19) used for the treatment of B-cell acute lymphoblastic leukemia is an effective and advanced bispecific antibody (Lim et al., 2021). The PD1/PD-L1 blocking antibody currently approved by the FDA shows a significant therapeutic activity, especially in patients with advanced NSCLC and melanoma. The combination of immune checkpoints with specific antigens on immune cells or tumor cells form bispecific antibodies is a promising approach (Bhandaru and Rotte, 2019). CSPG4 and MC1R are surface antigens often expressed on melanoma cells, and they participate in many biological behaviors of melanoma. Therefore, these two tumor-specific antigens are used as target antigens for the development of new antibodies and vaccines (Bluemel et al., 2010). They can form bispecific antibodies with immune checkpoint to achieve more precise targeting, which may increase the anti-tumor effect while reducing the side effects of immunotherapy. One of the key aspects of tumor drug resistance is to block the induction or conduction of cell death signals. Drugs directly induce cell death to break drug resistance, thus activating the death receptors expressed in melanoma cells to achieve effective therapeutic effects. The cell membrane resists the passage of macromolecular proteins, resulting in the inability of antibodies to bind to the internal antigens of tumor cells. Bispecific antibodies can be used as carriers on the one hand and be combined with intracellular antigens on the other hand, so as to achieve a wider range of biological effects produced by antigen-antibody binding.

## Future Outlooks/Challenges

Although bispecific antibodies are superior to monoclonal antibodies in terms of therapeutic effects, their structure is more complex, with large molecular weight and strong immunogenicity, which may induce the production of anti-drug antibodies (ADA) and lead to undesirable consequences. ADA can destroy or eliminate antibody activity by removing it from the circulation or blocking its function. The factors that determine the immunogenicity of bispecific antibodies are diverse and complex (Harding et al., 2010), and it is generally believed that immunogenicity is closely related to the homology of endogenous substances. The lower the homology of proteins to the corresponding endogenous counterpart, the higher its immunogenic potential (Husain and Ellerman, 2018). Co-administration of methotrexate and TNF-*α* antibody may reduce the rate of ADA formation in inflammatory bowel disease (Sasson et al., 2021). It has also been reported that avoiding antibiotic therapy may reduce the risk of ADA during anti-TNF therapy (Gorelik et al., 2021). However, reports on ADA treatment in cancer therapy are rare. Therefore, further studies are needed to reduce their immunogenicity to make bispecific antibodies with broader application prospects.

Furthermore, bispecific antibody fragments lacking the Fc region require continuous infusion to maintain a constant blood concentration because of their short half-life (Portell et al., 2013). Two popular bispecific antibody platforms successfully tried to add the Fc region to overcome this challenge. Another way to extend the half-life is to add albumin or albumin binding entities (Kontermann, 2011).

Cytokine release syndrome and neurotoxicity are the two main aspects of bispecific antibody toxicity. The first consists of the release of cytokines mediated by the activation of immune cells in the body, which is a systemic inflammatory response (Li et al., 2019), while the biological mechanism of neurotoxicity remains unclear (Stein et al., 2019). Both cytokine release syndrome and neurotoxicity are reduced by gradual administration of dexamethasone, administration of dexamethasone before the therapy, or temporary treatment discontinuation (Clynes and Desjarlais, 2019; Klinger et al., 2020). Our aim in the future is to explore more in-depth multivalent and multispecific antibodies, which could widen the way of bispecific antibodies on treating melanoma.
